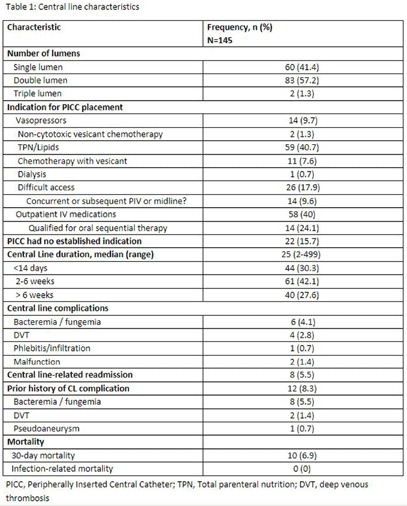# Opportunities to Reduce Peripherally Inserted Central Catheter (PICC) Utilization

**DOI:** 10.1017/ash.2025.295

**Published:** 2025-09-24

**Authors:** Rija Alvi, Anita Shallal, Geehan Suleyman

**Affiliations:** 1 Henry Ford Hospital; 2 Henry Ford Hospital; 3 Henry Ford Health

## Abstract

**Background:** Central line-associated bloodstream infections (CLABSIs) remain an important, preventable healthcare-associated infection. Prolonged catheterization is a major risk factor, and avoidance and prompt removal of unnecessary central lines (CL), including peripherally inserted central catheter (PICC), can reduce CLABSIs. We aimed to evaluate potential opportunities to reduce PICC utilization and associated harm. **Methods:** This was a cross-sectional observational study of hospitalized patients with PICCs from June 1 to June 30, 2024 at an 877-bed tertiary care hospital in Detroit. CL indications using evidence-based and institutional guidelines, duration of catheterization, and complications of line were evaluated. **Results:** 145 patients had PICCs (Table 1). Of these, 114 (78.6%) were placed at bedside in the general practice unit, 31 (21.3%) in the ICU and the majority (57.5%) were double lumen. Common indications included total parental nutrition (TPN) (59, 40.7%) and outpatient parenteral antimicrobial therapy (OPAT) (58, 40%). 22 (15%) patients did not have an established indication for PICC placement. Among patients receiving PICC for TPN, 9 (15%) did not meet criteria, and 9 (15%) were on TPN for < 5 days. Amongst those discharged on OPAT, 14 (24%) had opportunity for oral sequential therapy; 11 (19%) patients only received treatment for < 28 days. Although 26 (18%) patients had CL placed for difficult access, half of them had a concurrent or subsequent PIV or midline. Median duration of CL was 25 days (range: 2-499), and a third had CL placed for < 1 4 days. Overall, 22 (15.7%) patients were identified to not meet any indication for PICC and of those who received double or triple lumen catheter, 62 (73%) qualified for single lumen catheter only. Complications occurred in 13 (9%) patients, including CLABSI (6, 4.1%) and thrombotic events (4, 3%). Eight (5.5%) patients had line-related readmission. **Conclusion:** Upon review, PICC lines were commonly overutilized, and contributed to increased CLABSI rates. Several opportunities to reduce CLABSIs were identified, including reinforcement of appropriate CL indications, increase midline utilization for shorter duration of therapy and difficult access. These findings also encourage use of oral sequential therapy instead of OPAT, and placement of single lumen catheters where indicated.

**Figure:**